# Licorice attenuates cisplatin-induced hepatotoxicity by alleviating endoplasmic reticulum stress and apoptosis

**DOI:** 10.3389/fphar.2025.1557125

**Published:** 2025-03-20

**Authors:** Jie Li, Xiujuan Yang, Xiaolong Lian, Baojian Li, Quhuan Ma, Lingling Yang, Guangmiao Gao, Yi Deng, Zhijun Yang

**Affiliations:** ^1^ School of Pharmaceutical Science, Gansu University of Traditional Chinese Medicine, Lanzhou, Gansu, China; ^2^ Department of pharmacy, Qinghai University Medical College, Xining, China; ^3^ Shaanxi University of Chinese Medicine, Xi’an, China

**Keywords:** licorice, hepatotoxicity, ER stress, PERK/ATF4/CHOP pathway, apoptosis

## Abstract

Cisplatin (CP), a widely used antineoplastic drug, could induce hepatotoxicity and is also one of the most common reasons for drug-induced liver injury (DILI). Licorice (Chinese name GanCao, GC) is a commonly used herbal drug in traditional Chinese medicine (TCM) that has been shown to treat liver diseases and DILI. CP has been documented to induce apoptosis through the promotion of endoplasmic reticulum (ER) stress. However, the exact role of ER stress in the pathogenesis of CP-induced hepatotoxicity remains unclear. A rat DILI model was constructed through intraperitoneal injection of CP, and the anti-DILI effect of GC was detected by liver coefficients, liver function tests, pathological staining, and oxidative stress indices. Additionally, the ER stress and apoptosis indices were investigated by quantitative real-time PCR (qRT-PCR), Western blotting, and immunofluorescence (IF) on CP-induced toxicity in rat liver tissues and LO2 cells. In the model group, liver function indicators significantly elevated, liver lesions more pronounced, and the reactive oxygen species (ROS) level in the liver increased, the expression of ER stress markers, apoptosis factors, and indicators related to the protein kinase RNA-like ER kinase/activating transcription factor 4/C/EBP homologous protein (PERK/ATF4/CHOP) pathway significantly elevated. Treatment of the CP-induced toxicity in the rat model with GC significantly improved liver function, reduced liver lesions, decreased liver ROS. In addition, GC significantly inhibited the expression of ER stress markers, apoptosis factors, and indicators related to PERK/ATF4/CHOP pathway, demonstrating the anti-CP-induced hepatotoxicity effect of GC. In this study, we verified the protective effect of GC in CP-induced hepatotoxicity in rats and clarified its mechanisms related to ER stress and apoptosis.

## Introduction

Drug-induced liver injury (DILI), which is a liver disorder resulting from prescription and non-prescription drugs, is a major contributor to acute liver failure and death worldwide (Fernandez-Checa et al., 2021). To date, more than 1,000 drugs have been associated with DILI (Segovia-Zafra et al., 2021). Cisplatin (CP) is a widely used antitumor drug; however, severe hepatotoxic side effects limit its efficacy (Abd Rashid et al., 2021). Cisplatin induced hepatotoxicity is currently a major health problem due to its unpredictability and potentially fatal outcome, which can lead to acute liver failure or death in severe cases (Bademci et al., 2021). In addition, the hepatotoxicity of cisplatin can further aggravate renal toxicity (Galfetti et al., 2020). However, the potential mechanism of cisplatin induced liver injury has not been fully studied. Data suggest that the pathogenesis of CP-induced liver injury is thought to be a multi-factorial process involving inflammatory response, oxidative stress injury, endoplasmic reticulum (ER) stress, and apoptosis, ultimately leading to hepatocyte death (Aboraya et al., 2022; Fathy et al., 2022; Gad El-Hak et al., 2022). Consequently, elucidation of the mechanisms of hepatocyte death is essential for developing protective therapeutic interventions against CP-induced hepatotoxicity.

Oxidative stress serves as a significant mechanism of CP-induced hepatotoxicity, and many studies have indicated that oxidative stress imbalance is closely associated with the pathogenesis of CP-induced liver injury (Abd Rashid et al., 2021). Reactive oxygen species (ROS) are by-products of aerobic metabolism, which can lead to oxidative stress imbalance during drug-induced liver injury. Meanwhile, the production of ROS can lead to mitochondrial dysfunction or inflammation, which accelerates ER dysfunction and induces the unfolded protein response (UPR), which ultimately triggers apoptosis in hepatocytes (Villanueva-Paz et al., 2021). Studies have shown that oxidative stress caused by the interaction between CP and ER cytochrome P450 leads to apoptosis through activation of caspase12 (Liu and Baliga, 2005). Apoptosis, a primary death-signaling response, can occur through intrinsic, extrinsic, or ER stress pathways (Ajoolabady et al., 2023). Activation of ER stress often leads to hepatocyte injury and death (Wu et al., 2021). When drug treatment leads to the accumulation of misfolded proteins in the lumen of the ER, the UPR is activated in response to stress, promoting inflammation, oxidative stress, and apoptosis. However, as ER stress increases, apoptotic pathways are activated, ultimately leading to hepatocyte death (Huang et al., 2023). Research has found that drugs that inhibit GRK2 (CP-25 or paroxetine) significantly improve CP-induced acute liver injury (ALI) by suppressing oxidative stress, ER stress, and reducing hepatocyte apoptosis (Wang et al., 2024). In addition, Yu et al. found that acupuncture can improve CP-induced liver injury through reducing the expression of p-IRE-1α, GRP78 and caspase-12, and inhibiting the apoptosis rate of liver cells to reduce ER stress and apoptosis (Yu et al., 2024). These findings suggest a likely interrelated mechanism of ROS overproduction leading to oxidative stress imbalance, sustained activation of apoptosis signaling pathways and the onset of ER stress, adding to the complexity of the pathophysiology of DILI. Therefore, it is clinically important to explore prevention and treatment strategies that simultaneously aim at oxidative stress, ER stress and apoptosis to ameliorate CP-induced liver injury.

PERK is a type I transmembrane protein located on the ER membrane. Excessive activation of PERK signaling may impede a long-term protein translation, upregulate CHOP transcription factors, suppress anti-apoptotic genes expression and enhance pro-apoptotic genes expression, thereby accelerating cell death (Wang J. et al., 2022). PERK signaling pathway plays a crucial role in the mechanism of cisplatin toxicity. Tang Z et al. found that IS enhances cisplatin induced apoptosis by activating the PERK-eIF2α-ATF4-CHOP pathway mediated by ER stress (Tang et al., 2022). Li X et al. demonstrated that cisplatin activates transcriptional activity mediated by ER stress response elements in hepatoma cells by increasing the mRNA level of PERK pathway (Li et al., 2018). Ertunc O et al. found that IRB alleviates cisplatin induced hepatotoxicity by inhibiting ER stress by reducing the gene expression of the downstream PERK factor CHOP (Ertunç et al., 2024). These studies indicate that PERK is closely related to ER stress-mediated apoptosis in the mechanism induced by CP. Consequently, the PERK pathway is a promising therapeutic target for alleviating CP-induced ER stress and apoptosis.

Studies have shown that natural extracts with multiple biological activities have promising potential in preventing and treating various liver injuries, such as bergamot juice extract (Lombardo et al., 2024), Cardamom Extract (Sun L. et al., 2022), Jasminum grandiflorum L. extract (Alkhalifah et al., 2022), Olive phenolic compounds (Maalej et al., 2017), etc. Natural medicines are one of the important resources for the development of medicine, Licorice (Chinese name GanCao, GC) is derived from the dried root of *Glycyrrhiza uralensis* Fisch. ex DC., which was first described in Shen Nong Ben Cao Jing. GC has been extensively utilized on account of its anti-inflammatory, antiviral and antioxidant attributes. GC and its pharmacologically active constituents, such as glycyrrhetinate, glycyrrhizic acid, and licoaryl-coumarin, alleviate the symptoms of drug- and tox-in-induced poisoning and reduce mortality (Li et al., 2019). Our previous study showed that GC may ameliorate CP-induced hepatotoxicity by regulating bile acid metabolism and gut microbiota. However, the molecular mechanism of GC in the treatment of CP-induced liver injury remains unclear. It has previously been shown that elevated CP-induced ROS levels lead to an imbalance in oxidative stress, which further leads to ER stress and promotes tissue damage (Demir et al., 2024). Furthermore, the active constituents of GC have been reported to attenuate ER stress-induced hepatocyte apoptosis by modulating the CHOP/DR5/Caspase-8 pathway in cholestasis (Zou et al., 2023). Therefore, we hypothesized that GC might prevent CP-induced hepatocyte apoptosis by inhibiting ROS generation, alleviating oxidative stress injury and reducing ER stress. Thus, this study aimed to determine the mechanism of GC modulation of ER stress and apoptosis during CP-induced hepatotoxicity.

## Materials and methods

### GC sample preparation

Wild roots of *G. uralensis* Fisch. ex DC., purchased from Jinta, Jiuquan (Gansu, China), were identified by Prof. Yi Deng (Gansu University of Chinese Medicine). The extraction of Wild GC powder was conducted by employing 70% ethanol, and the reflux process was performed twice, each lasting for 3 h. Subsequently, the filtrates were merged, concentrated, and ultimately freeze-dried to form powders (39.6% yield). The lyophilized powder was stored at 4°C and diluted into different concentrations with normal saline before the experiments.

### Analysis and identification of blood absorption components of GC

Using UPLC-Q-TOF-MS/MS technique, 57 compounds including terpenoids, phenols, amino acids and other metabolites of GC were identified after GC administration in serum, blank serum and GC extract.

### CP-induced model and drug administration

Male SD rats were randomly separated into the following six groups (8 rats per group): (1) Control, (2) CP (8 mg/kg), (3) CP + Silibinin (44.1 mg/kg), (4) CP + GC (225 mg/kg), (5) CP + GC (450 mg/kg), and (6) CP + GC (900 mg/kg) (Li et al., 2024). GC, suspended in normal saline, and silibinin was used as the positive control group (purity ≥ 98%; Aladdin, Shanghai, China), suspended in 0.3% CMC-Na, were administered once a day for eight consecutive days. The control group and model group rats were given an equal volume of physiological saline via gavage, while the silibinin group and GC group were given drugs via gavage. On the fifth day, 30 min after gavage administration, apart from the control group, rats were intraperitoneally injected with CP (purity ≥ 98%; Solarbio, Beijing, China) at 8 mg/kg to induce liver injury (Li et al., 2024). After the last dose of normal saline, silibinin, and GC, the rats were fasted for 24 h, and then anesthetized by intraperitoneal injection of 30% pentobarbital sodium (800 mg/kg). Blood was withdrawn from the abdominal aorta, and the serum was stored at −20°C for later use. Then, the rats were euthanized using pentobarbital sodium, and the liver tissue was flushed with normal saline, frozen at −80°C. Liver function biochemical indicators and oxidative stress indicators were all performed using serum samples. Hematoxylin and eosin (HE) staining, terminal deoxynucleotidyl transferase-mediated 2′-deoxyuridine 5′-triphosphate (dUTP)-biotin nick end labeling (TUNEL) staining, dihydroethidium (DHE) staining, and immunofluorescence (IF) staining were all performed using one leaf of liver tissue. The ER ultrastructure, Western blotting (WB), and quantitative real-time PCR (qRT-PCR) indicators were all detected using the same leaf of liver tissue.

### Histological analysis

Pathological changes were observed with HE staining (kits: hematoxylin (CR2311076, Servicebio) and eosin (CR2402037-5, Servicebio)), and apoptosis of liver was observed with TUNEL staining (kit: G1504-50T, Servicebio). Images were analyzed with Case-Viewer2.3. The degree of liver injury is assessed using a four-level grading system called the liver score, which primarily involves a composite scoring of three pathological indicators, including hepatocyte edema, necrosis, and inflammatory cell infiltration. The scoring criteria are as follows: 1) Normal range: 0 points; 2) Very mild: 1 point; 3) Mild: 2 points; 4) Moderate: 3 points; 5) Severe: 4 points. Three fields are randomly selected from each section. For IF (kit: GDP1011, Servicebio) analysis, tissue sections were fixed with ice acetone for 20 min and then incubated in phosphate buffered saline (PBS) containing 5% bovine serum albumin (BSA) for 1 h. Subsequently, incubation was carried out at 4°C with primary antibodies against ATF4 (1:200, BM5179, Boster) and CHOP (1:500, 15204-1-AP, Proteintech). The slides were incubated with secondary antibodies for 1 h. The nuclei were counterstained with 4′,6-diamidino-2-phenylindole (DAPI). The results were observed using a fluorescence microscope.

### Biochemical testing

The serum levels of aspartate aminotransferase (AST) (B9F140223003X06801, Min-dray), total bilirubin (TBIL) (A3H140623002X08431, Mindray), alanine aminotransferase (ALT) (B9E140123004X03617, Mindray), and lactate dehydrogenase (LDH) (B9J142723002X03276, Mindray) were assessed by the automatic biochemical analyzer (Beckman Coulter, Shanghai, China).

### Determination of ROS generation

Fresh excised livers were dabbed dry using filter paper, followed by immediate freezing in a cryosectioning medium (OCT, Servicebio, Wuhan, China). Post-sectioning, the tissue was placed on anti-dehiscence slides, and a working solution of dihydroethidium DHE staining (100 µL) (kit: 38483-26-0, Aladdin) was dripped gradually. The sections were immediately incubated at 37°C for 40 min, protected from light. Upon sealing the sections with glycerol gelatine, they were examined under a fluorescence microscope (Leica DM6000M, Germany).

### Ultrastructural observation of the ER

Liver tissue was fixed in 4% glutaraldehyde, washed with cacodylate buffer, and fixed in 1% osmium tetraoxide. Subsequently, the tissues were dehydrated and embedded in epoxy resin. Ultra-thin sections were stained with uranyl acetate and lead citrate. Results were detected using transmission electron microscopy (TEM).

### Cell culture and treatment

LO2 cells (cell number corresponds to CVCL_6926), procured from iCell Bioscience Inc. (Shanghai, China), were cultured in 1640 medium containing 20% fetal bovine serum and 1% penicillin-streptomycin. The cells were incubated at 37°C with 5% CO2 for 24 h to achieve optimal density, ensuring 80% confluence for all cell studies as per the experimental design. LO2 cells were treated with CP for 24 h to induce liver injury. GC (at concentrations of 0.625, 1.25, 2.5 μg/mL), silibinin (0.125 μg/mL), and 4-PBA (1.5 μg/mL) (ER stress inhibitor, purity ≥ 98%, Sigma, St Louis, MO, United States) were administered 4 h before modeling.

### Cell viability analysis

Cell viability was determined using the 3-(4,5-Dimethylthiazol-2-yl)-2,5-diphenyltetrazoliumbromide (MTT) (1334GR001, Biofroxx) method according to the manufacturer’s instructions. Briefly, LO2 cells were cultured in 96-well plates for 24 h and then treated with different doses of GC (0.625, 1.25, 2.5, 5 μg/mL) for 4 h. LO2 cells were then incubated with CP (1, 2, 4 μg/mL) for 24 h. 10 μL of MTT reagent was added to each well and incubated at 37°C for 2 h. The optical density was measured at 570 nm with an enzyme marker, and the formula for calculating cell viability is as follows:

Cell viability (%) = (OD value of dosing group - OD value of zeroing group)/(OD value of control group - OD value of zeroing group) × 100%

### Flow cytometric detection

Apoptosis was detected through staining Annexin V-APC (KGA1030, Keygen). LO2 cells were seeded at a density of 1 × 105 cells per well and cultured in a six-well plate. The cells were then washed with pre-cooled PBS buffer, suspended in binding buffer, and stained with PI and A-V reagents. Finally, the samples were detected by flow cytometry using excitation wavelengths of 488 and 535 nm. Each group of experiments was repeated 3 times. The cell apoptosis rate was analyzed using CytExpert software, and the results were expressed as the average apoptosis rate.

### WB analysis

WB analysis was performed as previously described (Sun L. et al., 2022) using specific primary antibodies against CHOP (1:1000, 15204-1-AP, Proteintech), ATF4 (1:1000, BM5179, Boster), ATF6 (1:1000, A00655, Boster), GRP78 (1:1000, BA 2042, Boster), p-IRE1α (1:1000, NB100-2323, Novus), p-eIF2α (1:1000, BM3942, Boster), Bcl-2 (1:1000, A00040-1, Boster), Bax (1:1000, A00183, Boster), cleaved caspase-3 (1:1000, #9664T, Cell Signaling Technology), caspase-12 (1:1000, BA3142, Boster), cleaved caspase-8 (1:5000, ab108333, Abcam), p-PERK (1:1000, abs137056, Absin) and β-actin (1:5000, 20536-1-AP, Proteintech). Using HRP-conjugated Rabbit Anti-Goat IgG (H + L) (1:1000, SA0000-1-4, Proteintech) as the secondary antibody.

### qRT-PCR analysis

Use the M5 Universal RNA Mini Kit (MF036-01, Mei5, Beijing, China) to extract total RNA from liver tissue and cultured hepatocytes. M5 Sprint qPCR RT Kit with gDNA Remover (MF949-T, Mei5, Beijing, China) was utilized for reverse transcription to generate cDNA. The 2X M5 HiPer SYBR Premix EsTaq kit (MF787-T, Mei5, China) was used for qRT-PCR. Relative quantitative analysis is performed using the 2^−ΔΔCT^ method. Primer sequences of CHOP, GRP78, ATF4, ATF6, caspase-8, and caspase-12 were synthesized by Servicebio Technology Co., Ltd. (Wuhan, China) in Table 1.

**TABLE 1:** Gene primer sequences.

Gene name	Forward primer (5’→3′)	Reverse primer (5’→3′)
Rat CHOP	AAT​TGG​GGG​CAC​CTA​TAT​CTC​ATC	GGC​TTT​GGG​AGG​TGC​TTG​T
Rat ATF4	TGG​CTA​TGG​ATG​GGT​TGG​TC	GCT​CAT​CTG​GCA​TGG​TTT​CC
Rat ATF6	CCA​GAG​GCT​CAA​AGT​CCC​AAG​T	TGA​CAT​GGA​GGT​GGA​GGG​ATA​T
Rat GRP78	CTC​ATC​GGA​CGC​ACT​TGG​A	CTC​GGC​AGT​TTC​CTT​CAT​TTT​A
Rat Caspase-8	AAG​AAC​TGG​CTG​CCC​TCA​AG	GCA​TAA​CCC​TGT​AGG​CAG​AAA​CC
Rat Caspase-12	GGA​GGT​AAA​TGT​TGG​AGT​GGC	TTG​TTG​CAG​ATG​ATG​AGG​GC
Human ATF6	CAG​TAC​CAA​CGC​TTA​TGC​CAT​T	TGT​AGG​ACA​GGT​TTA​GTC​ACG​GA
Human GRP78	ACC​GCT​GAG​GCT​TAT​TTG​GG	CTG​CCG​TAG​GCT​CGT​TGA​T
Rat β-actin	TGC​TAT​GTT​GCC​CTA​GAC​TTC​G	GTT​GGC​ATA​GAG​GTC​TTT​ACG​G
Human β-actin	CAC​CCA​GCA​CAA​TGA​AGA​TCA​AGA​T	CAT​CGT​GAA​GAA​CAT​CTG​GCT​C

### Statistical analysis

All experimental data were statistically analyzed using GraphPad Prism 9.0 software. One-way ANOVA followed by LSD *post hoc* test was used to test for differences between groups. *p*-values <0.05 were considered significant. All manuscripts reporting statistical analyses should follow the SAMPL guidelines.

## Results

### GC alleviates liver injury in CP-induced rats

**FIGURE 1:**
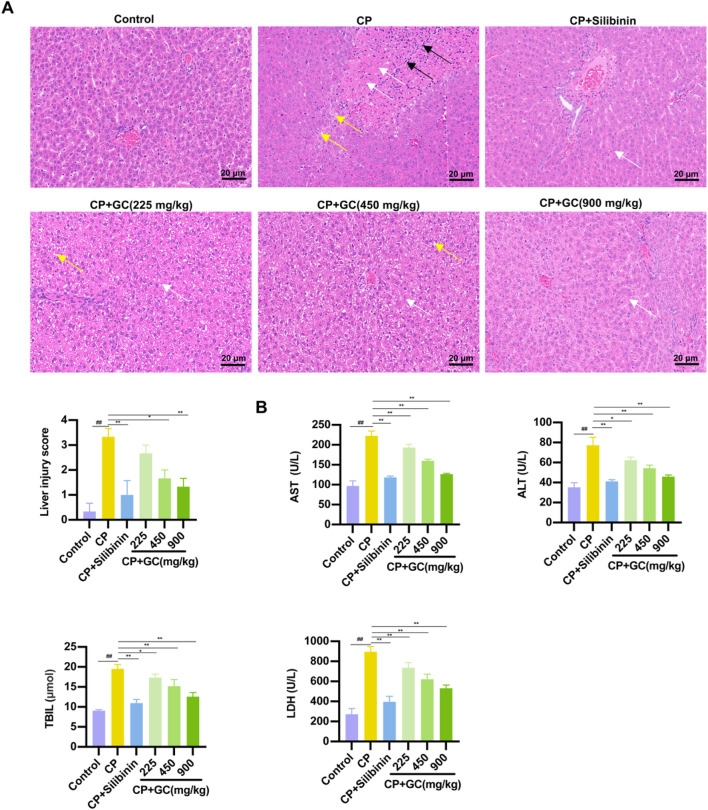
GC could ameliorate CP-induced liver injury. **(A)** Histological staining of liver tissue (400×) and histological staining of liver tissue for histological scoring. White arrows indicate liver cell necrosis, yellow arrows indicate liver cell edema, and black arrows indicate inflammatory cells. **(B)** Liver function parameters, including serum AST, ALT, TIBL, and LDH levels. All data are expressed as mean ± SD. ^##^ *p* < 0.01 compared with control group; ^*^ *p* < 0.05, ^**^ *p* < 0.01 compared with CP group.

Firstly, the pathological changes in liver tissue and the biochemical indicators of liver function (ALT, AST, TBIL, LDH) were evaluated. H&E staining results showed that the liver tissue in the CP group exhibited obvious edema, necrosis, and extensive infiltration of inflammatory cells. Silibinin and GC treatment effectively ameliorated CP-induced histological changes in the liver (Figure 1A). Furthermore, GC significantly ameliorated the liver injury and conspicuously decreased the liver histological score (Figure 1A). The result showed that serum LDH, AST, TBIL and ALT, which were significantly increased in the CP group, which were substantially reduced with GC treatment (Figure 1B). These results suggest that GC plays a protective role in CP-induced liver injury.

### GC inhibits CP-induced oxidative damage in rats

**FIGURE 2:**
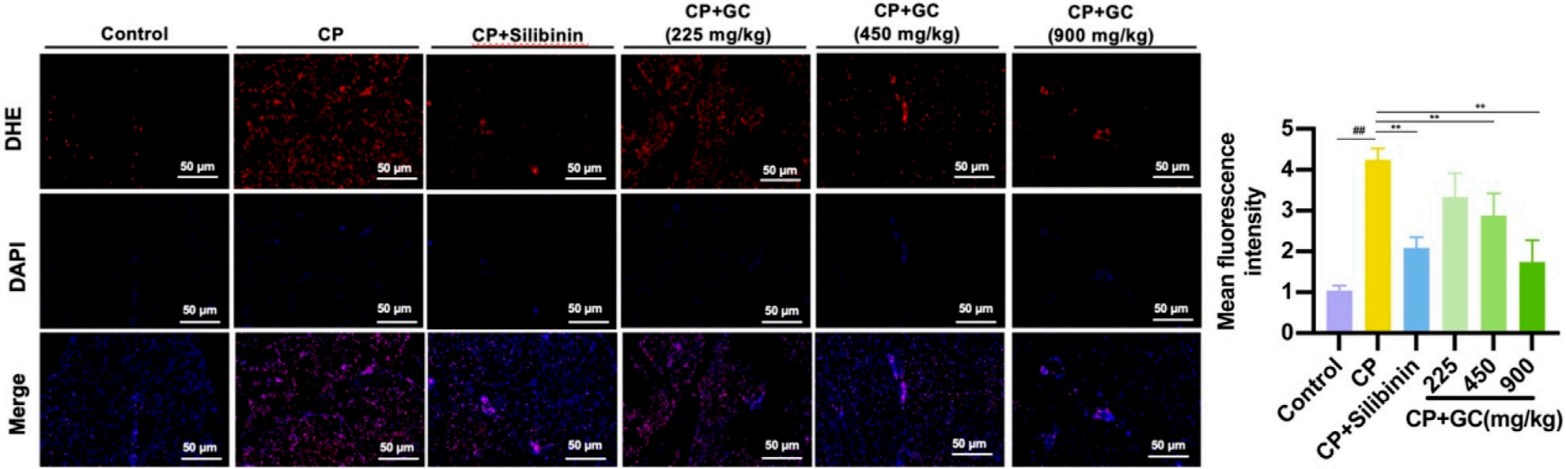
GC inhibits CP-induced oxidative damage in rats. Representative image and quantification of the DHE fluorescence. All data are expressed as mean ± SD. ^##^ *p* < 0.01 compared with control group; ^*^ *p* < 0.05, ^**^ *p* < 0.01 compared with CP group.

To assess the level of oxidative stress, ROS production in the livers of CP-induced hepatotoxic rats was measured by DHE staining. In rats with CP-induced hepatotoxicity, the quantity of DHE-stained cells was significantly elevated in liver tissue compared to controls. GC and silibinin treatment markedly decreased the quantity of DHE-stained cells in rat livers (Figure 2). The results show that GC could reduce CP-induced oxidative damage by inhibiting ROS generation.

### GC attenuates ER stress and apoptosis in CP-induced rats

**FIGURE 3:**
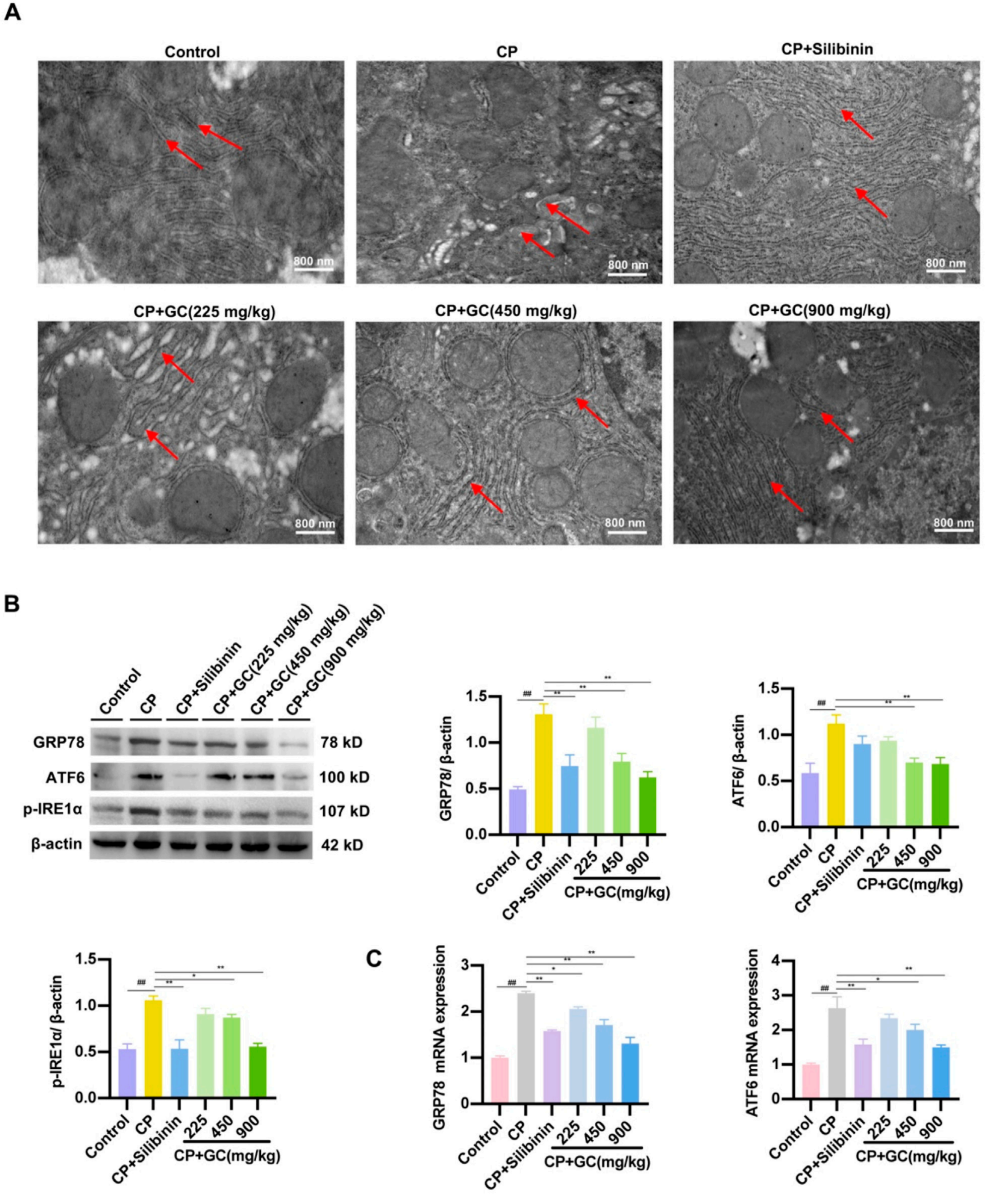
Effects of GC on CP-induced ER stress. **(A)** Effect of GC on the morphological changes of ER in liver cells induced by CP. Red arrows show the morphology of the endoplasmic reticulum and ribosomes. **(B, C)** Effect of GC on CP-induced ER stress-related indicators. GRP78, ATF6, and p-IRE1α protein expression were detected by WB analysis. GRP78 and ATF6 gene expression were detected by qRT-PCR analysis. All data are presented as the mean ± SD. ^##^ *p* < 0.01 compared with control group; ^*^ *p* < 0.05, ^**^ *p* < 0.01 compared with CP group.

To analyze the effect of GC on CP-induced ER stress, the ultrastructure of hepatocyte ER was observed via TEM. In the control group, liver cells were observed with parallel ER arranged, with black particles indicating ribosomes present as rough ER. In contrast, in the CP group, the cavity between the ER on either side of the liver cells was enlarged, indicating ER swelling, and the color of the ER became lighter, indicating the loss of black particles as smooth ER (Figure 3A). Additionally, key markers of ER stress were further detected using qRT-PCR and WB analysis. GC reduced the protein expressions of GRP78, ATF6, and p-IRE1α compared to the CP group (Figure 3B). Additionally, the gene ex-pressions of GRP78 and ATF6 was consistent with the results above (Figure 3C). In addition, we investigated whether GC inhibited CP-induced ER stress in rat liver. Not surprisingly, silibinin and GC had the same effect on CP-induced ER stress, reducing the CP-induced increase in the expressions of GRP78, ATF6 and p-IRE1α proteins and GRP78 and ATF6 mRNA. These findings indicate that CP-induced liver injury induces an ER stress response. Furthermore, GC and silibinin significantly inhibit CP-induced ER stress, thereby reducing hepatotoxicity.

**FIGURE 4:**
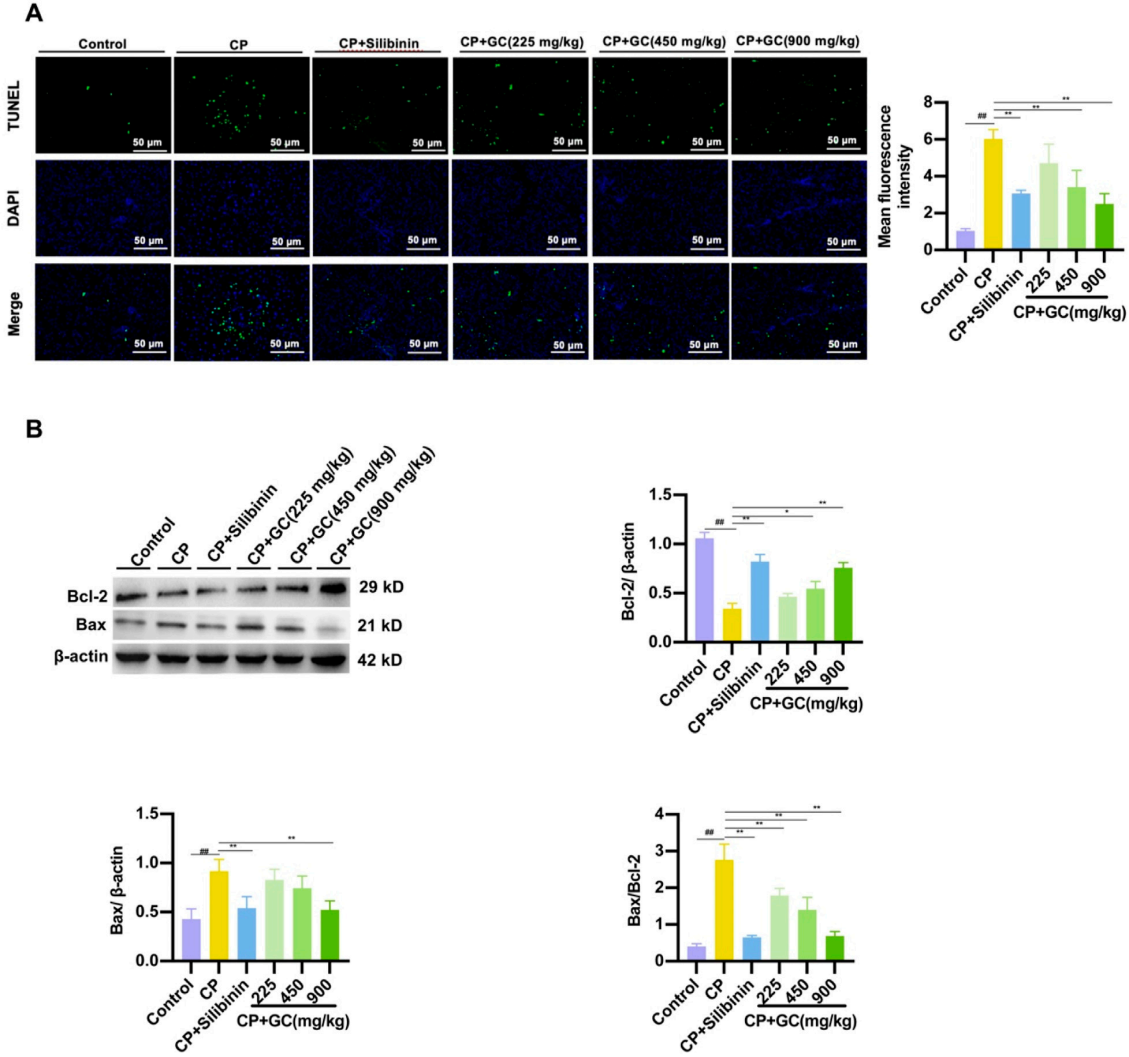
Effect of GC on CP-induced apoptosis levels in the liver. **(A)** Representative image and quantification of TUNEL staining. **(B)** Effects of GC on CP-induced apoptosis-related proteins in the liver. Protein expressions of Bax and Bcl-2 was assessed using WB. All data are presented as the mean ± SD. ^##^ *p* < 0.01 compared with control group; ^*^ *p* < 0.05, ^**^ *p* < 0.01 compared with CP group.

Next, the effect of GC on CP-induced apoptosis was evaluated by analyzing TUNEL staining and apoptotic protein expression. TUNEL staining (Figure 4A) indicated that GC treatment notably decreased CP-induced apoptosis in TUNEL-positive cells. In addition, CP-induced liver injury significantly upregulated Bax expression while downregulating the expression of Bcl-2. GC intervention significantly decreased the expression of Bax protein while increasing the expression of Bcl-2 protein (Figure 4B). Unsurprisingly, silibinin pretreatment had the same effect on the expression of apoptotic proteins (Figure 4B). The data confirmed that GC pretreatment reduced CP-induced apoptosis in the liver.

### GC attenuates CP-induced ER stress in LO2 cells

**FIGURE 5:**
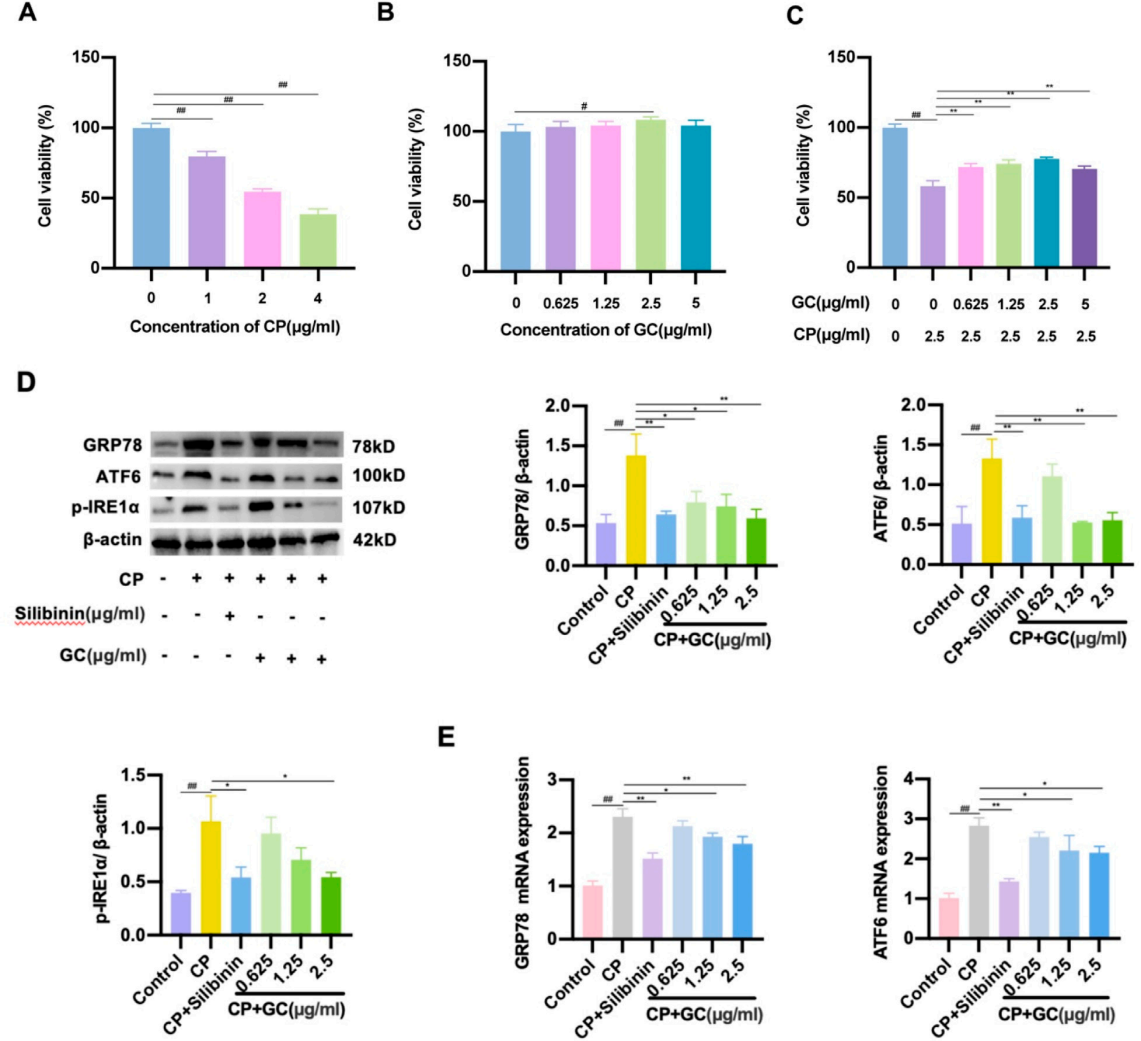
**(A-C)** MTT assay for determining cell viability. **(D-E)** GC reduced the expression of ER stress-related indicators. Expression levels of GRP78, ATF6, and p-IRE1α protein were tested by WB analysis. Expression levels of GRP78 and ATF6 mRNA were tested by qRT-PCR analysis.

To further validate the protective effect of GC against CP-induced liver injury after GC, a CP-treated DILI-LO2 hepatocyte model was established to investigate the effect of GC on ER stress. The effects of CP and GC on LO2 cell viability were determined by MTT. As displayed in Figure 5A, the viability of LO2 cells gradually decreased with increasing CP concentration. At a CP concentration of 2.5 μg/mL, the viability of LO2 cells was ap-proximately 50% (Figure 5A), which was chosen as the concentration for the subsequent hepatotoxicity test. Furthermore, GC had no cytotoxic effect on LO2 cells in the concentration range of 0–5 μg/mL (Figure 5B). Compared with the CP group, the cell survival rate was significantly increased after pretreatment of LO2 cells with GC (Figure 5C), suggesting that GC could protect injured LO2 cells. To assess the level of ER stress, we examined the expression of several key ER stress markers, GRP78, ATF6, and p-IRE1α in CP-induced LO2 cells by WB and qRT-PCR. GC treatment significantly reduced these markers at protein levels compared to the model group (Figure 5D). The trend of GRP78 and ATF6 mRNA expression is consistent with the results of WB (Figure 5E). Similarly, silibinin significantly alleviated the degree of CP-induced ER stress in LO2 cells (Figures 5D,E). In conclusion, GC can alleviate CP-induced hepatotoxicity by inhibiting ER stress.

### GC reduces CP-induced apoptosis in LO2 cells by suppressing ER stress

**FIGURE 6:**
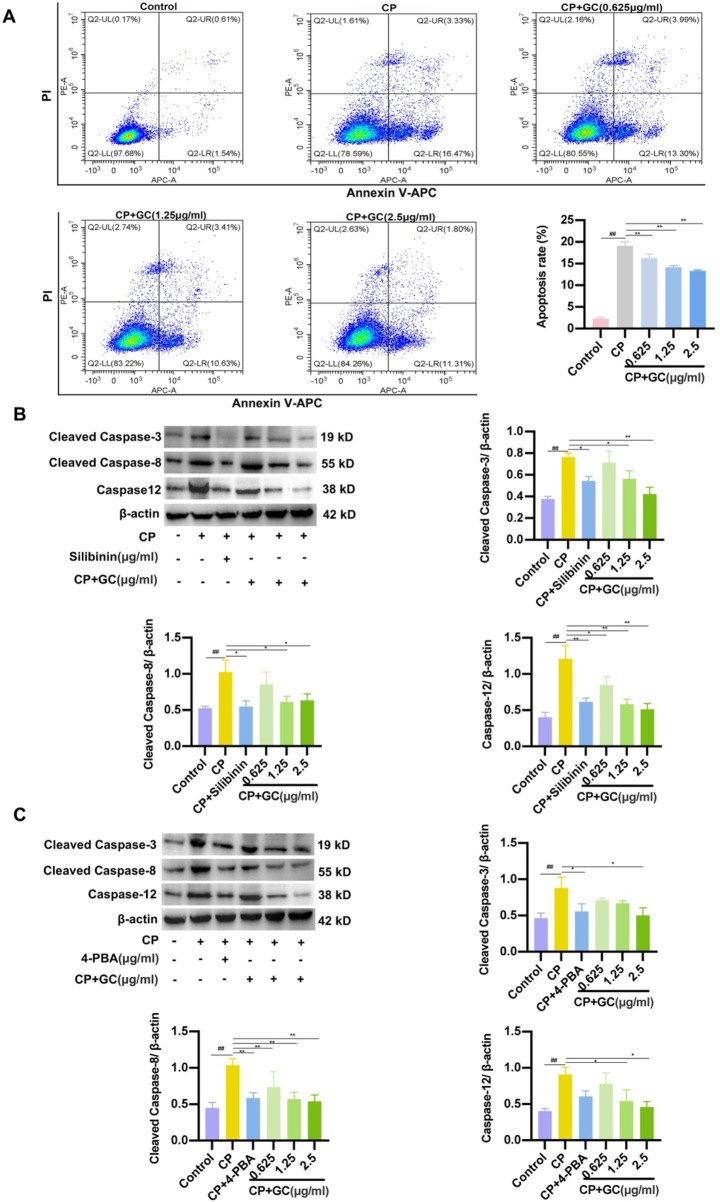
GC reduces CP-induced apoptosis in LO2 cells by suppressing ER stress. **(A)** Flow cytometry results of apoptosis in LO2 cells. **(B)** Effects of GC and Silibinin on CP-induced apoptosis-related proteins. **(C)** Effect of 4-PBA on CP-induced apoptosis-associated proteins. WB analysis was performed to detect the expressions of caspase-12, cleaved caspase-3 and cleaved caspase-8. All data are expressed as mean ± SD. ^##^ *p* < 0.01 compared with control group; ^*^ *p* < 0.05, ^**^ *p* < 0.01 compared with CP group.

Flow cytometry analysis was performed to assess the level of apoptosis in LO2 cells, and GC treatment significantly reduced the proportion of apoptotic cells compared to the CP group (Figure 6A). In addition, WB analysis were used to detect the expressions of cleaved caspase-3, cleaved caspase-8 and caspase-12 at the protein level in rats. As presented in Figure 6B, the expression of apoptotic proteins was significantly increased in CP-induced LO2 cells, whereas GC and silibinin decreased the expression of these apoptotic proteins. The above results verified that administration of GC and silibinin prevented CP-induced apoptosis. Interestingly, we found that pretreatment with 4-PBA (ER stress inhibitor) decreased the expression of these apoptotic proteins (Figure 6C). Accordingly, by reducing ER stress, GC may reduce CP-induced apoptosis in LO2 cells.

### GC attenuated CP-induced apoptosis by modulating the ER-mediated PERK/ATF4/CHOP pathway

**FIGURE 7:**
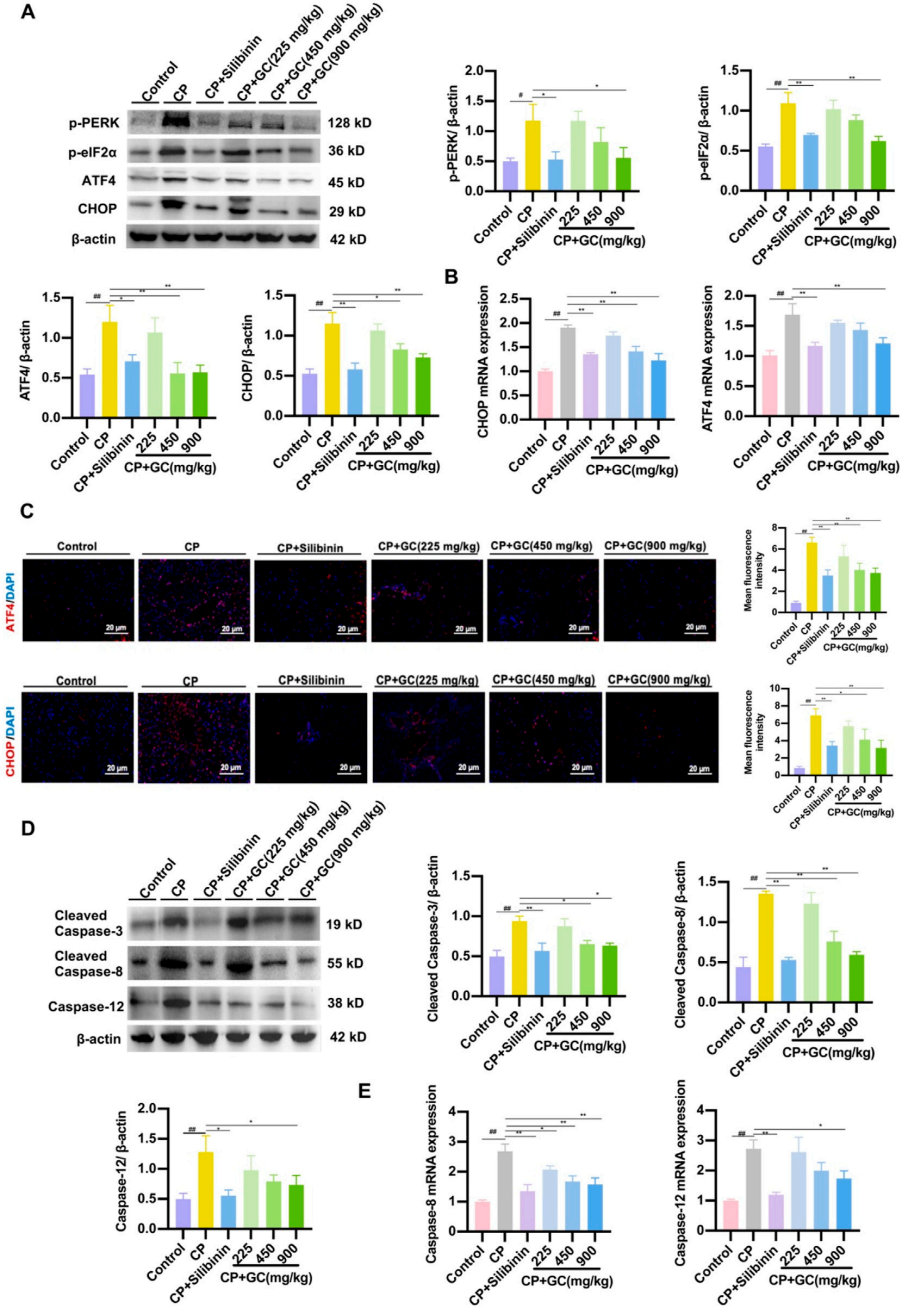
GC attenuated CP-induced apoptosis by modulating the ER-mediated PERK/ATF4/CHOP pathway. **(A)** The expressions of p-PERK, p-eIF2α, ATF4 and CHOP proteins were detected by WB analysis. **(B)** qRT-PCR was performed to detect the expression of CHOP and ATF4 mRNA. **(C)** Representative image and quantification of the immunofluorescence of CHOP and ATF4. **(D)** The expressions of cleaved caspase-3, cleaved caspase-8, and caspase-12 proteins were detected by WB analysis. **(E)** qRT-PCR was performed to detect the expression of caspase-8, and caspase-12 mRNA. All data are presented as the mean ± SD. ^##^ *p* < 0.01 compared with control group; ^*^ *p* < 0.05, ^**^ *p* < 0.01 compared with CP group.

To further explore the specific mechanism of ER stress-induced apoptosis, we hypothesized that the molecular mechanism by which GC prevents hepatocyte CP-induced apoptosis may be associated with the PERK/ATF4/CHOP signaling pathway. As illustrated in Figure 7A, protein expressions of ATF4, p-PERK, CHOP and p-eIF2α were significantly increased in the liver of the CP group. However, GC and silibinin effectively inhibited the expressions of ATF4, p-PERK, CHOP and p-eIF2α and further inhibited the expression of the apoptotic factor CHOP. PCR assays and immunofluorescence observations were consistent with the above results (Figures 7B, C), suggesting that GC could antagonize ER stress to achieve anti-hepatotoxicity effects. Besides, protein expressions of apoptosis-related factors (cleaved caspase-3, cleaved caspase-8, and caspase-12) down-stream of CHOP was significantly reduced in the GC-treated group compared to the CP-treated group (Figure 7D). PCR assays were consistent with the above results (Figure 7E). The results showed that CP-mediated hepatotoxicity induced hepatocyte apoptosis by stimulating ER stress and further activating PERK/ATF4/CHOP signaling in the liver. In contrast, GC and silibinin suppressed hepatocyte apoptosis by alleviating CP-induced ER stress by inhibiting the PERK/ATF4/CHOP pathway.

## Discussion

ER stress is closely associated with various drug-induced liver dysfunctions. Apoptosis is among the most significant types of cell death in DILI (Sun Y. et al., 2022). Consequently, preventing ER stress and hepatocyte apoptosis may be the key to treating DILI. This study found that GC may attenuate CP-induced oxidative stress and further attenuate CP-induced ER stress, thereby inhibiting hepatocyte apoptosis. The underlying mechanisms of these effects may be related to the regulation of PERK/ATF4/CHOP-related signaling pathways.

Oxidative stress homeostasis is crucial in CP-induced hepatotoxicity (Okkay et al., 2022). ROS production is a significant contributor to CP-induced oxidative injury. We found that GC suppressed the generation of ROS when compared with the model group. This suggests that GC reduced oxidative damage to the liver due to CP-induced toxicity. Furthermore, silibinin, an antioxidant flavonoid complex from milk thistle (silybum marianum), inhibits oxidative stress and attenuates CP-induced hepatotoxicity (Ahmed, 2024). Not surprisingly, our results showed that silibinin inhibited ROS production induced by CP. Silibinin and GC shared the same effect on CP-induced oxidative stress suppression. These outcomes imply that the mechanism against CP-induced hepatotoxicity is linked to the antioxidant properties of GC.

ER stress is a significant factor in hepatocyte injury and plays a crucial role in the pathogenesis of DILI (Pu et al., 2023). The accumulation of misfolded proteins mediated by the ER due to stimuli such as viral and pharmacological agents is often implicated as the cause of ER stress (Wang N. et al., 2022). To maintain ER homeostasis and reduce stress, the UPR can be initiated. However, UPR overactivation due to sustained or severe ER stress can yield cellular damage and death (Baral, 2024). GRP78—a key receptor protein for ER stress—regulates ER transmembrane transactivation through UPR activation (Chuan et al., 2021). ATF6, IRE1α and PERK are ER transmembrane receptor proteins that can activate three signaling pathways down-stream of the UPR. Activation of ER stress and UPR leads to the binding of GRP78 to unfolded proteins, causing separation from ER transmembrane receptors. This results in the autophosphorylation of IRE1α and PERK, while ATF6 is activated via protein hydrolysis (Ong et al., 2024). Furthermore, the activation of the UPR signaling pathway affects genes that improve ER stress (Ong et al., 2024). Tsai et al. found that glycyrrhetinic acid reduced ER stress-related protein levels and ameliorated acute liver injury associated with total parenteral nutrition in rats (Tsai et al., 2013). In this study, the expressions of p-IRE1α, GRP78 and ATF6 were increased in CP-induced liver tissue and LO2 cells. GC significantly reduces these factors in both liver tissue and liver cells. To investigate whether GC inhibited CP-induced ER stress in rat liver and whether it was associated with ROS. we pretreated CP-induced rat and LO2 cells with silibinin. The data indicated that silibinin reduced the expressions of p-IRE1α, GRP78 and ATF6 by inhibiting ROS generation. Silibinin and GC had the same effect on CP-induced ER stress inhibition. These findings suggest that GC plays a protective role in liver function by inhibiting CP-induced ER stress and is associated with ROS.

In this study, CP significantly increased Bax/Bcl-2 protein expression in liver tissues and cleaved caspase-3, cleaved caspase-8, and caspase-12 protein expressions in LO2 cells. However, GC reduced the expression of these apoptotic proteins. The TUNEL assay results showed that GC significantly reduced the number of TUNEL-positive cells compared to the CP group. Additionally, flow cytometry results revealed that CP in-creased the percentage of apoptotic cells in LO2 hepatocytes, whereas GC treatment significantly decreased the percentage of apoptotic cells. In light of these findings, we postulated that GC could suppress apoptosis induced by CP. Apoptosis is a pathway mediated by intrinsic apoptosis, extrinsic apoptosis, or ER stress (Ajoolabady et al., 2023). ER-mediated apoptosis-associated proteins CHOP and caspase-12 can induce apoptosis. CHOP is an ER stress transducer and a key pro-apoptotic signaling protein that downregulates Bcl-2 expression, upregulates Bax expression, and induces caspase-8 activation, which further activates downstream caspase-3, leading to apoptosis (Zou et al., 2023). Caspase-12 is a protein enzyme activated by IRE1α and associated with ER-mediated cell apoptosis. Excessive ER stress triggers the caspase-12 response, which promotes the progression of cell death by activating the caspase-3-induced apoptotic mechanism (Murata et al., 2024). Therefore, we hypothesized that GC could prevent apoptosis by reducing ER stress. In order to verify this assumption, we pre-treated LO2 cells with 4-PBA (ER stress inhibitor) and silibinin. The results showed that 4-PBA and silibinin had the same effect as GC in attenuating CP-induced apoptosis in LO2 cells. This suggests that GC may reduce apoptosis by inhibiting oxidative stress and ER stress during CP-induced liver injury.

The PERK/ATF4/CHOP signaling pathway plays a key role in ER stress-induced liver disease (Hou et al., 2022). EIF2α is a significant substrate of PERK; its phosphorylation leads to the arrest of protein translation, thereby maintaining ER homeostasis (Huang et al., 2024); CHOP has been identified as a key factor in ER stress-induced apoptosis, and sustained PERK signaling can enhance stress-specific ATF4 expression, thereby inducing upregulation of CHOP expression (Wu et al., 2023). Studies have shown that modulating PERK pathway can prevent DILI (Chen et al., 2022). For example, rosiglitazone can alleviate APAP-induced ALI by down-regulating protein levels of p-PERK, p-eIF2α and CHOP in the PERK pathway and attenuating ER stress (Cao et al., 2022). In addition, CAT inhibits ER stress by down-regulating the expression of p-PERK, ATF4, and CHOP, thereby reversing TP-induced hepatotoxicity (Zhang et al., 2022). Similar to these results, our results found that GC mitigated CP-induced hepatotoxicity by reducing protein expression of p-PERK, p-eIF2α, ATF4, and CHOP in the PERK pathway. Apoptosis is closely related to PERK/ATF4/CHOP pathway mediated by ER stress. Studies have shown that LPS can inhibit the activation of caspase-12 and caspase-3 by inhibiting the ATF4-CHOP pathway to reduce hepatocyte apoptosis (Rao et al., 2013). Similarly, this study found that GC treatment downregulated the expression of apoptosis-related factors downstream of CHOP (caspase-12, cleaved caspase-3, and cleaved caspase-8), suggesting that GC mitigated CP-induced apoptosis by inhibiting ER stress. This is consistent with the results of *in vitro* analysis. Therefore, we hypothesize that GC attenuates CP-induced hepatocyte apoptosis by inhibiting ER stress-mediated PERK/ATF4/CHOP signaling pathway, thereby alleviating hepatotoxicity.

Overall, based on the experiments *in vivo* and *in vitro*, this study revealed the hepatotoxic protective mechanism of GC against CP from the perspective of ER stress and apoptosis, providing some new insights for the prevention of CP-induced hepatotoxicity. Although the findings of this study are of great significance, there are still some limitations that need to be further explored in future studies. Firstly, on the basis of cell experiments, it is necessary to further elucidate the influence mechanism of ER stress and apoptosis in CP-induced liver injury through ER stress inhibitor treatment in animal studies. Second, in addition to the PERK pathway, this study also needs to further clarify the effects of GC on the ER stress-mediated ATF6 and IRE1α pathways in the mechanism of CP hepatotoxicity.

## Conclusion

This study found that GC exerts a protective role in CP-induced liver toxicity. Animal studies showed that GC reduced ROS level in the liver to inhibit oxidative stress induced by CP. Furthermore, GC reversed the expression of ER stress markers (GRP78, ATF6, and p-IRE1α) and cell apoptosis-related factors (Bax/Bcl-2, cleaved caspase-3, cleaved caspase-8, and caspase-12) that were elevated, and inhibited the PERK/ATF4/CHOP pathway, indicating that GC inhibits CP-induced ER stress to reduce cell apoptosis in liver tissue and thus protect liver function. Similarly, cell experiments showed that GC alleviated ER stress in CP-induced hepatocytes and inhibited hepatocyte apoptosis, demonstrating the anti-CP hepatotoxicity of GC. Therefore, the protective mechanism of GC against CP-induced liver toxicity may be related to inhibiting ER stress and apoptosis.

## Data Availability

The raw data supporting the conclusions of this article will be made available by the authors, without undue reservation.

## Abbreviations

ALT, alanine aminotransferase; AST, aspartate aminotransferase; ATF4, activating transcription factor 4; ATF6, activating transcription factor 6; Bcl2, b-cell lymphoma 2; BSA, bovine serum albumin; BPI, base peak ion; CHOP, C/EBP homologous protein; CP, cisplatin; DAPI, 4′,6-diamidino-2-phenylindole; DILI, drug-induced liver injury; eIF2α, eukaryotic initiation factor 2α; ELISA, enzyme-linked immunosorbent assay; ER stress, endoplasmic reticulum stress; GRP78, glucose-regulated protein 78; HE, hematoxylin and eosin; IRE1α, inositol-requiring en-zyme-1α; LDH, lactate dehydrogenase; LT, labile enterotoxin; PBS, phosphate buffered saline; PERK, protein kinase RNA-like ER kinase; PI, propidium iodide; qRT-PCR, quantitative real-time PCR; RCN1, reticu-localbin 1; TBIL, total bilirubin; TCM, traditional Chinese medicine; TEM, transmission electron microscopy; TUNEL, terminal deoxynucleotidyl transferase-mediated dUTP-biotin nick end labeling; UPR, unfolded protein response; WB, Western blotting.

## References

[B1] Abd Rashid N. Abd Halim S. A. S. Teoh S. L. Budin S. B. Hussan F. Ridzuan N. R. A. (2021). The role of natural antioxidants in cisplatin-induced hepatotoxicity. J. Biomed. and Pharmacother. 144, 112328. 10.1016/j.biopha.2021.112328 34653753

[B2] Aboraya D. M. El, Baz A. Risha E. F. Abdelhamid F. M. (2022). Hesperidin ameliorates cisplatin induced hepatotoxicity and attenuates oxidative damage, cell apoptosis, and inflammation in rats. J. Saudi J. Biol. Sci. 29, 3157–3166. 10.1016/j.sjbs.2022.01.052 35844386 PMC9280168

[B3] Ahmed M. (2024). A therapeutic and protective effect of silymarin against hepatotoxicity induced by cisplatin in female rats. Tikrit J. Agric. Sci. 24 (3), 74–84. 10.25130/tjas.24.3.7

[B4] Ajoolabady A. Kaplowitz N. Lebeaupin C. Kroemer G. Kaufman R. J. Malhi H. (2023). Endoplasmic reticulum stress in liver diseases. J. Hepatol. 77, 619–639. 10.1002/hep.32562 PMC9637239 35524448

[B5] Alkhalifah E. A. R. Alobaid A. A. Almajed M. A. Alomair M. K. Alabduladheem L. S. Al-Subaie S. F. (2022). Cardamom extract alleviates the oxidative stress, inflammation and apoptosis induced during acetaminophen-induced hepatic toxicity via modulating Nrf2/HO-1/NQO-1 pathway. Curr. Issues Mol. Biol. 44 (11), 5390–5404. 10.3390/cimb44110365 36354677 PMC9688982

[B6] Bademci R. Erdoğan M. A. Eroğlu E. Meral A. Erdoğan A. Atasoy Ö. (2021). Demonstration of the protective effect of ghrelin in the livers of rats with cisplatin toxicity. Hum. and Exp. Toxicol. 40 (12), 2178–2187. 10.1177/09603271211026722 34151639

[B7] Baral A. (2024). Endoplasmic reticulum stress signaling in the regulation of hepatic pathological responses. Stresses 4 (3), 481–504. 10.3390/stresses4030031

[B8] Cao Y. He W. Li X. Huang J. Wang J. (2022). Rosiglitazone protects against acetaminophen-induced acute liver injury by inhibiting multiple endoplasmic reticulum stress pathways. J. BioMed Res. Int. 10, 6098592. 10.1155/2022/6098592 PMC9797312 36588533

[B9] Chen J. Wu H. Tang X. Chen L. (2022). 4-Phenylbutyrate protects against rifampin-induced liver injury via regulating MRP2 ubiquitination through inhibiting endoplasmic reticulum stress. Bioengineered 13 (2), 2866–2877. 10.1080/21655979.2021.2024970 35045794 PMC8974152

[B10] Chuan L. Huang X. Fan C. Wen S. Yang X. Wang J. (2021). Metformin ameliorates brain damage caused by cardiopulmonary resuscitation via targeting endoplasmic reticulum stress-related proteins GRP78 and XBP1. J. Eur. J. Pharmacol. 891, 173716. 10.1016/j.ejphar.2020.173716 33197442

[B11] Demir S. Mentese A. Usta Z. T. Alemdar N. T. Demir E. A. Aliyazicioglu Y. (2024). Alpha-pinene neutralizes cisplatin-induced reproductive toxicity in male rats through activation of Nrf2 pathway. J. Int. Urology Nephrol. 56, 527–537. 10.1007/s11255-023-03817-5 37789204

[B12] Ertunç O. Erzurumlu Y. Savran M. Çataklı D. Doğan Kıran E. Pekgöz Ş. (2024). Potential hepatoprotective effects of irbesartan, an accessible angiotensin II receptor blocker, against cisplatin-induced liver injury in a rat model. Turkish J. Pharm. Sci. 21 (2), 88–94. 10.4274/tjps.galenos.2023.90846 PMC11096784 38742755

[B13] Fathy M. Darwish M. A. Abdelhamid A. S. M. Alrashedy G. M. Othman O. A. Naseem M. (2022). Kinetin ameliorates cisplatin-induced hepatotoxicity and lymphotoxicity via attenuating oxidative damage, cell apoptosis and inflammation in rats. J. Biomed. 10, 1620. 10.3390/biomedicines10071620 PMC9312964 35884925

[B14] Fernandez-Checa J. C. Bagnaninchi P. Ye H. Sancho-Bru P. Falcon-Perez J. M. Royo J. F. (2021). Advanced preclinical models for evaluation of drug-induced liver injury-consensus statement by the European Drug-Induced Liver Injury Network [PRO-EURO-DILI-NET]. J. J. Hepatology 75, 935–959. 10.1016/j.jhep.2021.06.021 34171436

[B15] Gad El-Hak H. N. Mahmoud H. S. Ahmed E. A. Elnegris H. M. Aldayel T. S. Abdelrazek H. M. A. (2022). Methanolic Phoenix dactylifera L. extract ameliorates cisplatin-induced hepatic injury in male rats. J. Nutr. 14, 1025. 10.3390/nu14051025 PMC8912432 35268000

[B16] Galfetti E. Cerutti A. Ghielmini M. Zucca E. Wannesson L. (2020). Risk factors for renal toxicity after inpatient cisplatin administration. BMC Pharmacol. Toxicol. 21, 1–7. 10.1186/s40360-020-0398-3 32122396 PMC7052961

[B17] Hou W. Nsengimana B. Yan C. Nashan B. Han S. (2022). Involvement of endoplasmic reticulum stress in rifampicin-induced liver injury. Front. Pharmacol. 13, 1022809. 10.3389/fphar.2022.1022809 36339603 PMC9630567

[B18] Huang M. Wang Y. Wu X. Li W. (2024). Crosstalk between endoplasmic reticulum stress and ferroptosis in Liver diseases. Front. Bioscience-Landmark 29 (6), 221. 10.31083/j.fbl2906221 38940044

[B19] Huang X. Zhu H. Lu W. Cao L. Fang Z. Che L. (2023). Acute endoplasmic reticulum stress suppresses hepatic gluconeogenesis by stimulating MAPK phosphatase 3 degradation. Int. J. Mol. Sci. 24 (21), 15561. 10.3390/ijms242115561 37958545 PMC10647389

[B20] Li J. Lian X. Li B. Ma Q. Yang L. Gao G. (2024). Pharmacodynamic material basis of licorice and mechanisms of modulating bile acid metabolism and gut microbiota in cisplatin-induced liver injury based on LC-MS and network pharmacology analysis. J. J. Ethnopharmacol. 340, 119293. 10.1016/j.jep.2024.119293 39736346

[B21] Li X. Pan E. Zhu J. Xu L. Chen X. Li J. (2018). Cisplatin enhances hepatitis B virus replication and PGC-1α expression through endoplasmic reticulum stress. Sci. Rep. 8 (1), 3496. 10.1038/s41598-018-21847-3 29472690 PMC5823916

[B22] Li X. Sun R. Liu R. (2019). Natural products in licorice for the therapy of liver diseases: progress and future opportunities. J. Pharmacol. Res. 144, 210–226. 10.1016/j.phrs.2019.04.025 31022523

[B23] Liu H. Baliga R. (2005). Endoplasmic reticulum stress–associated caspase 12 mediates cisplatin-induced LLC-PK1 cell apoptosis. J. Am. Soc. Nephrol. 16 (7), 1985–1992. 10.1681/ASN.2004090768 15901768

[B24] Lombardo G. E. Navarra M. Cremonini E. (2024). A flavonoid-rich extract of bergamot juice improves high-fat diet-induced intestinal permeability and associated hepatic damage in mice. Food and Funct. 15 (19), 9941–9953. 10.1039/d4fo02538e 39263833

[B25] Maalej A. Mahmoudi A. Bouallagui Z. Fki I. Marrekchi R. Sayadi S. (2017). Olive phenolic compounds attenuate deltamethrin-induced liver and kidney toxicity through regulating oxidative stress, inflammation and apoptosis. Food Chem. Toxicol. 106, 455–465. 10.1016/j.fct.2017.06.010 28595958

[B26] Murata N. Nishimura K. Harada N. Kitakaze T. Yoshihara E. Inui H. (2024). Insulin reduces endoplasmic reticulum stress‐induced apoptosis by decreasing mitochondrial hyperpolarization and caspase‐12 in INS‐1 pancreatic β‐cells. Physiol. Rep. 12 (12), e16106. 10.14814/phy2.16106 38884322 PMC11181300

[B27] Okkay I. Okkay U. Aydin I. C. Bayram C. Ertugrul M. S. Mendil A. S. (2022). *Centella asiatica* extract protects against cisplatin-induced hepatotoxicity via targeting oxidative stress, inflammation, and apoptosis. J. Environ. Sci. Pollut. Res. 29, 33774–33784. 10.1007/s11356-022-18626-z 35029831

[B28] Ong G. Ragetli R. Mnich K. Doble B. W. Kammouni W. Logue S. E. (2024). IRE1 signaling increases PERK expression during chronic ER stress. Cell Death and Dis. 15 (4), 276. 10.1038/s41419-024-06663-0 PMC11026449 38637497

[B29] Pu S. Pan Y. Zhang Q. You T. Yue T. Zhang Y. (2023). Endoplasmic reticulum stress and mitochondrial stress in drug-induced liver injury. Molecules 28 (7), 3160. 10.3390/molecules28073160 37049925 PMC10095764

[B30] Rao J. Qin J. Qian X. Lu L. Wang P. Wu Z. (2013). Lipopolysaccharide preconditioning protects hepatocytes from ischemia/reperfusion injury (IRI) through inhibiting ATF4-CHOP pathway in mice. PLoS One 8 (6), e65568. 10.1371/journal.pone.0065568 23750267 PMC3672158

[B31] Segovia-Zafra A. Zeo-Sánchez D. E. López-Gómez C. Pérez-Valdés Z. García-Fuentes E. Andrade R. J. (2021). Preclinical models of idiosyncratic drug-induced liver injury (iDILI): moving towards prediction. J. Acta Pharm. Sin. B 11, 3685–3726. 10.1016/j.apsb.2021.11.013 PMC8727925 35024301

[B32] Sun L. Zhang Y. Wen S. Li Q. Chen R. Lai X. (2022a). Extract of Jasminum grandiflorum L. alleviates CCl4-induced liver injury by decreasing inflammation, oxidative stress and hepatic CYP2E1 expression in mice. Biomed. and Pharmacother. 152, 113255. 10.1016/j.biopha.2022.113255 35689859

[B33] Sun Y. Zhang Y. Xie L. Rong F. Zhu X. Xie J. (2022b). Progress in the treatment of drug-induced liver injury with natural products. J. Pharmacol. Res. 183, 106361. 10.1016/j.phrs.2022.106361 35882295

[B34] Tang Z. Du W. Xu F. Sun X. Chen W. Cui J. (2022). Icariside II enhances cisplatin-induced apoptosis by promoting endoplasmic reticulum stress signalling in non-small cell lung cancer cells. Int. J. Biol. Sci. 18 (5), 2060–2074. 10.7150/ijbs.66630 35342361 PMC8935239

[B35] Tsai J. J. Kuo H. C. Lee K. F. Tsai T. H. (2013). Glycyrrhizin represses total parenteral nutrition-associated acute liver in-jury in rats by suppressing endoplasmic reticulum stress. J. Int. J. Mol. Sci. 14, 12563–12580. 10.3390/ijms140612563 23771023 PMC3709800

[B36] Villanueva-Paz M. Morán L. López-Alcántara N. Freixo C. Andrade R. J. Lucena M. I. (2021). Oxidative stress in drug-induced liver injury (DILI): from mechanisms to biomarkers for use in clinical practice. J. Antioxidants 10, 390. 10.3390/antiox10030390 PMC8000729 33807700

[B37] Wang J. Ding L. Wang K. Huang R. Yu W. Yan B. (2022a). Role of endoplasmic reticulum stress in cadmium-induced hepatocyte apoptosis and the protective effect of quercetin. J. Ecotoxicol. Environ. Saf. 241, 113772. 10.1016/j.ecoenv.2022.113772 35714484

[B38] Wang N. Wang H. Zhang J. Ji X. Su H. Liu J. (2022b). Endogenous peroxynitrite activated fluorescent probe for revealing anti-tuberculosis drug induced hepatotoxicity. Chin. Chem. Lett. 33, 1584–1588. 10.1016/j.cclet.2021.09.046

[B39] Wang Q. Li M. Duan F. Xiao K. Sun Q. Q. Cheng J. R. (2024). GRK2 mediates cisplatin-induced acute liver injury via the modulation of NOX4. Cell Biol. Toxicol. 40 (1), 98. 10.1007/s10565-024-09940-y 39546067 PMC11567994

[B40] Wu H. Bao X. Gutierrez A. H. Nevzorova Y. A. Cubero F. J. (2023). Role of oxidative stress and endoplasmic reticulum stress in drug-induced liver injury. Explor. Dig. Dis. 2 (3), 83–99. 10.37349/edd.2023.00020

[B41] Wu J. Qiao S. Xiang Y. Cui M. Yao X. Lin R. (2021). Endoplasmic reticulum stress: multiple regulatory roles in hepatocellular carcinoma. J. Biomed. and Pharmacother. 142, 112005. 10.1016/j.biopha.2021.112005 34426262

[B42] Yu D. D. Wang J. M. Han L. DU X. Y. Chao L. Q. Zhang H. H. (2024). Mechanism of acupuncture and moxibustion in ameliorating liver injury induced by cisplatin by regulating IRE-1 signaling pathway. Zhen ci yan jiu= Acupunct. Res. 49 (7), 686–692. 10.13702/j.1000-0607.20240125 39020486

[B43] Zhang L. Li C. Fu L. Yu Z. Xu G. Zhou J. (2022). Protection of catalpol against triptolide-induced hepatotoxicity by inhibiting excessive autophagy via the PERK-ATF4-CHOP pathway. J. PeerJ 10, e12759. 10.7717/peerj.12759 PMC8742543 35036109

[B44] Zou B. Zhang S. Zhao J. Song G. Weng F. Xu X. (2023). Glycyrrhetinic acid attenuates endoplasmic reticulum stress-induced hepatocyte apoptosis via CHOP/DR5/Caspase8 pathway in cholestasis. J. Eur. J. Pharmacol. 961, 176193. 10.1016/j.ejphar.2023.176193 37981257

